# Reuse and Mechanochemical Processing of Ore Dressing Tailings Used for Extracting Pb and Zn

**DOI:** 10.3390/ma16217004

**Published:** 2023-11-01

**Authors:** Vladimir I. Golik, Roman V. Klyuev, Nikita V. Martyushev, Viktor V. Kondratiev, Vadim S. Tynchenko, Vitaliy A. Gladkikh, Liudmila V. Iushkova, Vladimir Brigida

**Affiliations:** 1 Department “Technique and Technology of Mining and Oil and Gas Production”, Moscow Polytechnic University, 33 B. Semenovskaya St., 107023 Moscow, Russia; v.i.golik@mail.ru (V.I.G.); kluev-roman@rambler.ru (R.V.K.); 2 Materials Science Department, Tomsk Polytechnic University, 30 Lenin Ave., 634050 Tomsk, Russia; 3 Laboratory of Geochemistry of Ore Formation and Geochemical Methods of Prospecting, A. P. Vinogradov Institute of Geochemistry of the Siberian Branch of the Russian Academy of Sciences, 664033 Irkutsk, Russia; 4 Department of Technological Machines and Equipment of Oil and Gas Complex, School of Petroleum and Natural Gas Engineering, Siberian Federal University, 660041 Krasnoyarsk, Russia; vadimond@mail.ru; 5 Information-Control Systems Department, Institute of Computer Science and Telecommunications, Reshetnev Siberian State University of Science and Technology, 660037 Krasnoyarsk, Russia; 6 Artificial Intelligence Technology Scientific and Education Center, Bauman Moscow State Technical University, 105005 Moscow, Russia; 7 Stroytest Research and Testing Center, Moscow State University of Civil Engineering, 26 Yaroslavskoye Shosse, 129337 Moscow, Russia; 8 Basic Department, Higher School of Restaurant Management, Siberian Federal University, 660041 Krasnoyarsk, Russia; 9 Department of State and Municipal Administration, Siberian Fire and Rescue Academy of State Fire Service of the Ministry of Emergency Situations of Russia, 662972 Zheleznogorsk, Russia; 10 Department of Biomedical, Veterinary and Ecological Directions, RUDN University, 6 Miklukho-Maklaya St., 117198 Moscow, Russia; 1z011@inbox.ru; 11 Research Institute of Comprehensive Exploitation of Mineral Resources of the Russian Academy of Sciences, 4 Kryukovskiy Tupik, 111020 Moscow, Russia

**Keywords:** hydrometallurgical process, chemical activation, leaching Pb, failure (mechanical), circular waste management, resource use efficiency, environmental management, sustainable production, heavy metals and pollution

## Abstract

The increasing accumulation of rock waste obtained due to ore processing and its environmental impacts, such as acid mine drainage and elevated concentrations of heavy metals in soils, necessitates the transformation of mining technologies based on the concept of circular waste management. The research is aimed at improving the parameters of the mechanical activation effect produced on technogenic georesources, as well as at expanding the application scope of disintegrators in the field of using the partial backfill of the mined-out space when developing stratified deposits. In this regard, the research purpose was to substantiate the parameters of extracting metals from enrichment tailings using their mechanochemical activation to ensure cyclic waste management. The research involved the application of three-dimensional interpolation methods used for processing the data and the graphical representation. As a result, the following was found to be characteristic of the waste of the Sadonsky mine management. The degree of extracting zinc from pre-activated tailings increases logarithmically when the H_2_SO_4_ concentration and the NaCl proportion decrease 3.5 times. The degree of extracting lead from the activated tailings increases according to the Fourier law when decreasing the NaCl mass concentration, and an optimal range of the H_2_SO_4_ (0.38–0.51%) proportion decreases six times. One of the key results of the research is the justification of expanding the scope of applying disintegrators in the case of a directed activation influence exerted on the components of the stowing strips. The obtained results expand the understanding of the mechanism of the influence of the mechanochemical activation of dry tailings on the reactivity unevenness when extracting several metals from them.

## 1. Introduction

At the present stage of socio-economic development, developed countries are facing global challenges in the field of consuming energy, water, and resources and ensuring sustainable economic growth [1,2,3]. The growing need for various metals while maintaining the natural environment quality necessitates the development of projects by mining enterprises in increasingly complex conditions, which causes the problem of resource endowment inequality [4,5,6,7]. The mining of energy resources and mineral raw materials becomes simultaneously a versatile source of solid and liquid wastes [8,9]. The volumes of the enrichment tailings in Chile alone reach 800 million t/year, while only 0.5 kg of metals can be extracted from each ton [10]. The increasing accumulation of wastes during ore dressing changes the geochemical composition of soils [11,12,13,14,15], which results in the fertility loss of the soils. In addition, significant threats to microbial communities are created [16] by changing the composition of soil gases [17,18]. The acid drainage processes [19,20] and the formation of salt crusts on horizontal sections of the terrain require an urgent improvement of the methodology of the integrated geo-ecological monitoring of the mining waste impact [21,22]. This aspect should be taken into account when implementing both promising geotechnologies [23,24] and methods of complex waste processing [25,26,27,28,29].

One of the advanced fields in this issue is forming “circular waste management” [30,31,32,33,34,35]. This system of measures is primarily aimed at optimizing material flows based on their cyclicity at all stages of production [36,37,38,39,40]. Another approach within the framework of the “circularity” concept is the re-extraction of metals from technogenic raw materials of old tailings [41,42] or after the direct formation of enrichment tailings [43,44,45], ash residue of solid household waste [46], and the reuse of various kinds of waste [47,48,49].

Leaching the metals is simulated mainly on the basis of the equations of the inverse exponential function [50], as well as other two-dimensional graphical representations (for example, during the atmospheric leaching of mixed chlorides) [51]. The presence of many exogenous factors influencing the leaching efficiency significantly complicates the formulation of the problem [52,53]. The most important factors are a ratio of H_2_SO_4_ and HCl in the leaching solution [54,55], a high-pressure value aimed at intensifying oxidative acid leaching [56,57], and leaching time [58,59,60]. Scientific works analysis [61,62,63,64] showed that in the metals leaching process, the following parameters are the most important: the lixiviant type (H_2_SO_4_, HCl, HNO_3_); stirring speed; solid-to-liquid (S/L) ratio; acid concentration; temperature; granulometric composition of tailings; leaching time. For example, with an S/L ≤ 20 g/L, the degree of leaching of rare earth elements is 50% higher than with an S/L = 100–200 g/L [65,66,67,68,69]. In most cases, sulfuric acid leaching is preferable for Zn extraction (Pb yield is minimal), while the use of hydrochloric acid can increase the Pb yield to 9.6% [55]. The degree of Pb extraction from tailings increases with increasing acid concentration from 1 M to 2 M (with leaching time = 48 h) when using H_2_SO_4_ from 46 to 58% and with HCl from 80 to 91% (which makes it more attractive for choice of acid) [67]. During sulfuric acid leaching of low-grade zinc-containing ores, a change in grinding fineness from −208 + 147 to −74 + 53 μm at 50 °C, 10% H_2_SO_4,_ and leaching time = 180 min leads to an increase in zinc yield from 28 to 80% [68]. Another similar study proved that a particle size of 75–80 µm is sufficient to recover 91.97% of zinc with a leaching time of only 20 min, 70 °C, and a sulfuric acid concentration of 100 g/L [69]. When using NaOH as a leaching solution, the grinding fineness had virtually no effect on the yield of Zn and Pb, amounting to 78 and 10%, respectively [70].

It is worth noting that the main problem of extracting metals from enrichment tailings is the search for optimal parameters and ways to increase their reactivity. The most promising direction for this is to ensure the amorphization of raw materials using a high-energy mechanical action [71,72]. The mechanochemical activation increases the surface area of a solid and decreases the coherence energy, causing a spontaneous aggregation, adsorption, or recrystallization of a geomaterial [73]. The general theory states that the main effect of high-energy grinding is achieved due to changes in the stress-strain and dispersion state, causing changes in the structure and chemical activity of the material. When processing the nickel ore (20% of H_2_SO_4_), the Ni concentration in the pulp varied from 88% to 98%; Co ranged from 96% to 98%; Fe varied from 82% to 90% when the leaching time was 60–120 min [74,75]. This study indicates the importance of assessing the mechanical activation effect of the tailings when the leaching time is minimal. At the same time, the assessment of the mutual influence of several factors exerted on leaching the metals remains insufficiently studied. Optimizing the ratio of the proportions of the reagents present in the leaching solution is also extremely important for the process under study. The kinetics of leaching Cu by the acid from activated chalcopyrite leads to the fact that the crystallinity degree of the geomaterial becomes 30% higher, and the dissolution rate increases by 40% [40,76]. The activation of siliceous tailings is known to change the pozzolanic activity unevenly when they are used as cement [77]. Therefore, the following question arises: can the mechanical activation effect cause an uneven effect of activating the leaching reactions when using different metals? In this connection, the research is aimed at testing the hypothesis consisting of the fact that when the agitation leaching time is short, the high-energy influence can cause a “competition” between metals for the consumption of lixiviants present in the leaching solution.

The concept of a closed cycle of geomaterials necessitates not only the disposal of waste but also its reuse in underground development [78,79,80]. The best thing is to produce declining stratified deposits in operating mines. For example, sodium sulfate that is used for activating slag pastes (filling mass) when the curing time is 28 days can reach 40 MPa [81,82,83].

In this regard, the purpose of the work is to substantiate the parameters of extracting metals from the enrichment tailings using their mechanochemical activation to ensure cyclic waste management.

## 2. Materials and Methods

The research object for the first task was the tailings of the Mizursky enriching factory (they have enriched pyrite–polymetallic ores since the 1970s) that represent a typical geomaterial of the Zgidsky, Sadonsky, and Archonsky deposits of the Sadonsky mining district of Russia. In addition, the volume of accumulated tailings already exceeds 4 million tons, and the chemical pollution zone is about 39.4 km^2^. The chemical composition of the raw materials is presented in Table 1, and the particle size is given in Table 2. For particles screened by a 0.1 mm sieve, the specific surface area was 129.2 m^2^/kg.

Similarly to [84], the general task of determining the metal yield was considered as a response function in the volume concentrations of lixiviants (h (H_2_SO_4_), g/L; h (NaCl), g/L); the ratio of solid and liquid fractions (S/L); the pulp mass (Mp); the agitation leaching time (t, min); the presence/absence of the activation influence; and the rotor rotation speed in the disintegrator (Speed, rpm), if available. The main parameters (ranges h of (H_2_SO_4_) h of (NaCl), S/L) were specially selected to be the same as those mentioned in the previous work and were assumed to be the same for each group of the experiments. As before, the five-dimensional formulation of the problem of determining the response space, specified implicitly, can be defined as Pb = ƒ(h; t; S/L; Mp). A detailed description of the formulas used for determining intermediate values during experiments is provided in [84].

The H_2_SO_4_-NaCl mixture is used in the technological process of the existing industry, and this determines the “basic version” parameters of the technology. The advantage of using NaCl is conditioned by the ability to extract Pb simultaneously with Zn by obtaining hydrochloric acid during the reaction between sodium chloride and sulfuric acid. The acid H_2_SO_4_ leaching is the “classic” and most effective option, although it is the least environmentally friendly. A more focused study in this area is [55].

**Figure 1:**
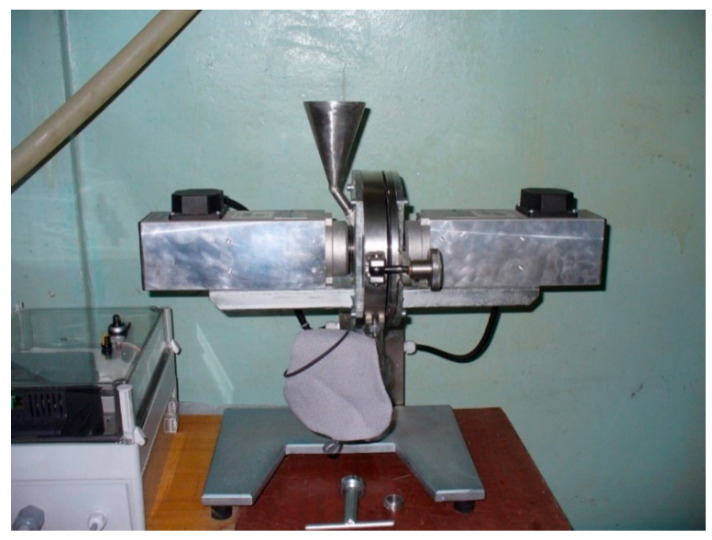
The laboratory installation intended for mechanical activation.

In this study, the first stage of the experiments required clarifying the influence of the preliminary dry mechanical activation effect (when the number of rotor rotations was minimum) exerted on the enrichment tailings during a minimum leaching time. The ground samples of the tailings were sieved through a 2.0 mm sieve to form the pulp. The activation effect on the pulp was produced by a DESI-11 disintegrator (Tootmise OÜ, Tallinn, Estonia) (Figure 1) at a rotor speed of 300 rpm and 1200 rpm, respectively, for 0.25 and 1 h.

The waste mass in each sample (3 repetitions) was 50 g. The concentration of the metals in the pulp was determined by a common technique using an atomic absorption spectrometer “KVANT-AFA” (OOO “KORTEK”, Moscow, Russia). Since the results of the experiments raised a number of questions when realizing the first stage, a decision was taken to conduct clarifying experiments on leaching zinc. To carry out percolation leaching, an ES-8400 overhead stirrer (Moscow, Russia) was used, while in all the experiments, the mixing speed was 50 rpm. In the second stage, the problem of assessing the influence of the disintegration effect on the longer leaching of Pb was solved (Table 3).

Table 3 shows that in the first set of the experiments “Li_Pb(0.25)”, the agitation leaching of lead from the geomaterial samples was conducted for 15 min. To consider a five-dimensional problem in several three-dimensional ones, in each set of experiments, two factors were assumed to be constant: the S/L and h(H_2_SO_4_)/h(NaCl) ratio. S/L took one of three values (1/4, 1/7 and 1/10). At the same time, the h(H_2_SO_4_)/h(NaCl) ratio was equal to the following series of values (2/20; 6/20; 10/20; 2/160; 6/160; 10/160; 6/90). In addition, to reduce the dimensionality, the Mp factor was completely neutralized by transiting to the mass concentration of lixivants present in it (m_P_ (H_2_SO_4_), % and m_P_ (NaCl), % (Table 4)).

The analysis of the scattered data remains a rather complex task that is solved in different ways. The algorithms based on machine learning or deep learning are mainly used [85,86,87], including ANN in combination with the Levenberg–Marquardt Scheme having backpropagation [88,89], Shapley Additive exPlanations (SHAP) in combination with CatBoost (an AI model was used for increasing the gradients on decision trees) [90], two-dimensional regression models [91,92,93], Shapley Value Regression [94], Nearest Neighbor Method [95], etc. The main disadvantages of ANN, as well as of other “stochastic” interpolation methods, are provided in [96,97,98].

Q-Q graphs were selected as a “goodness-of-fit” criterion used for checking the simulation quality. At the initial stage (as well as when constructing Q–Q graphs), the data were processed using the Microsoft Excel v2010 software. The author’s approach was based on the method of processing the experimental data, using the classical algorithm of “smoothing” the data in combination with the three-dimensional triangulation procedure of Renka R.J. [99,100], which were implemented in the form of “scripts” (using the Vi IMproved software (v9.0)), coded in Python (v2.7.10). The final three-dimensional graphs were constructed in the “gnuplot” software (v5.4). The data were regressed using the least square method intended for selecting the model parameters (“Scilab v6.1.1” software).

## 3. Results

### 3.1. The Influence of the Preliminary Dry Mechanical Activation Effect on the Agitation Leaching of Pb and Zn from Technogenic Raw Materials

The essence of the first stage of the research consisted of comparing two response surfaces: the agitation leaching of Pb during 15 min without activating the tailings (Li_Pb(0.25)), depending on different ratios of the mass fractions of H_2_SO_4_ and NaCl; leaching Pb from pretreated dry geomaterials in a disintegrator at a “speed” of 300 rpm (Des_Pb(0.25)) when the duration of the process and the ratios of the mass fractions of H_2_SO_4_ and NaCl are the same. In addition, at the first stage, an additional set of experiments on leaching Zn from the mechanically activated dry geomaterials at a “Speed” of 300 rpm (Des_Zn(0.25)) was carried out when the process duration and the ratio of lixivants were the same. The second stage of identifying the influence of the process duration on the efficiency of leaching Pb consisted of two sets of experiments, “Li_Pb(1)” and “Des_Pb(1)”, intended for the simple agitation leaching of Pb during 60 min and obtaining lead from the mechanically activated dry tailings at a “speed” of 1200 rpm. The results of all the above variants of the experiments are shown in Table 5.

**Figure 2:**
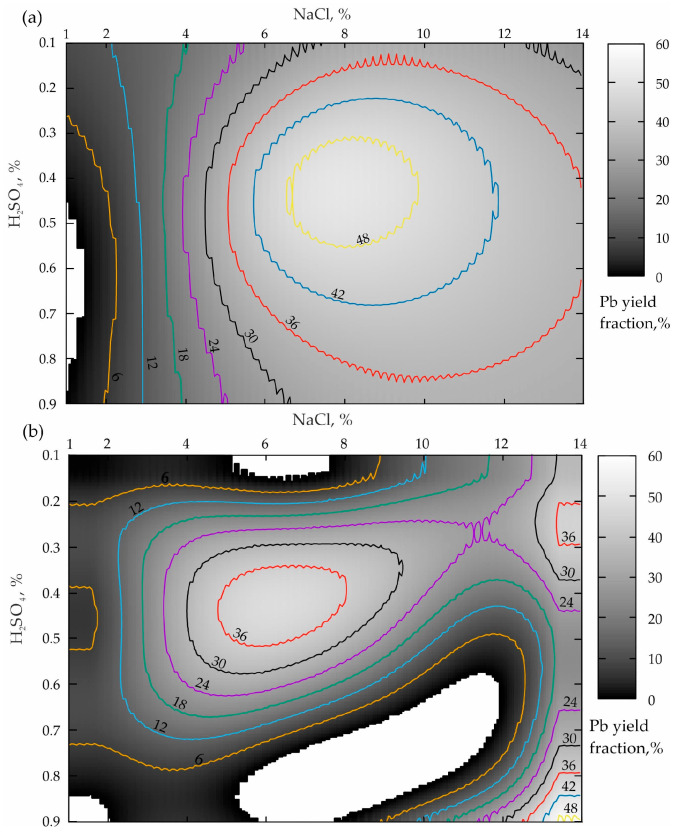
Distribution of the lead yield from the tailings of the Mizursky factory: (**a**)—leaching Pb without activating “Li_Pb(0.25)”; (**b**)—Pb yield from the pre-activated tailings Des_Pb(0.25).

The processing of the first two sets of the experiments when the leaching duration was 15 min, provided in Table 4, is demonstrated in Figure 2.

The analysis of the response surface projection shown in Figure 2a allows the conclusion that a NaCl concentration increase from 1 to 14% in the case of the boundary values of H_2_SO_4_ (0.1 and 0.9%) leads to an increase in the lead yield in the pulp from 6–12% to 30% according to a dependence that is close to the logarithmic one. To obtain the optimal concentration of the sulfuric acid (from 0.3 to 0.55%), starting with NaCl ≥ 1%, a monotonous increase in the Pb concentration from minimum values to a maximum of more than 48% in the NaCl range from 6.8 to 10% is also observed. When NaCl ≥ 10%, the Pb yield increase is replaced by a smooth decline to 38–36%. In this connection, a dependence by the type of a rational Taylor series was established for this “base case”, whose formula has the following form (R^2^ = 0.9):

(1) $$\mathrm{Pb}=\frac{\left(10.33-0.51\mathrm{NaCl}-37.64H_{2}{\mathrm{SO}}_{4}+0.08{\left(\mathrm{NaCl}\right)}^{2}+25.16{\left(H_{2}{\mathrm{SO}}_{4}\right)}^{2}+4.28{\mathrm{NaClH}}_{2}{\mathrm{SO}}_{4}\right)}{1-0.14\mathrm{NaCl}-2.10H_{2}{\mathrm{SO}}_{4}+0.01{\left(\mathrm{NaCl}\right)}^{2}+1.92{\left(H_{2}{\mathrm{SO}}_{4}\right)}^{2}+0.10{\mathrm{NaClH}}_{2}{\mathrm{SO}}_{4}},$$

The analysis results presented in Figure 2b demonstrate that the preliminary activation of the geomaterial leads to the response surface transformation accompanied by a pronounced tendency to shift the local maximum of the zone of the optimal ratio of lixivants to the left relatively NaCl, which is very good. At the same time, the value of the lead yield maximum decreased from 48 to 36%, which is a disadvantage. In addition, an insufficiently pronounced zone of the second local maximum (NaCl = 12–14%; H_2_SO_4_ = 0.65–0.9%) having a level of 48% appears, which corresponds to the base variant value. In view of this, the dependence in the form of the Fourier Series Bivariate Order 2 × 3 (R^2^ = 0.96) was established for this case of preliminary mechanical processing.

**Figure 3:**
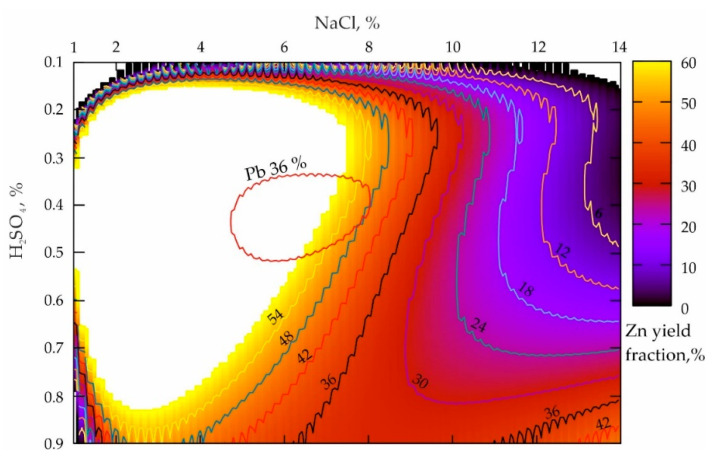
Distribution of the zinc yielded from the tailings of the Mizursky factory that were preliminary mechanically activated by the dry method.

A slight deterioration of leaching the lead from the technogenic geomaterials was difficult to predict; the only reason could be the high chemical activity of another metal, which was conditioned by the disintegrator action. To test the probability of the Zn influence on the lead yield reduction when the process parameters are optimal, a set of experiments, “Des_Zn(0.25)”, were conducted, whose results are shown in Figure 3.

The analysis of the response surface projection presented in Figure 3 allows the conclusion that the NaCl concentration increase from 1 to 14%, when the H_2_SO_4_ values were 0.1%, leads to a smooth decline in the zinc yield in the pulp from 54% to 6% (NaCl = 10.5%) and less according to a dependence that is close to the parabolic one. When the H_2_SO_4_ values are 0.9%, a NaCl proportion decrease results in a sharp decline in the zinc yield of up to about 30% (NaCl = 6.5–10%) and then in a slight increase of up to 42% when NaCl = 14%. A characteristic peculiarity of zinc is a very large local maximum zone, and its extreme left location along NaCl indicates a very high reactivity of Zn with respect to Pb (during their mechanical activation). Moreover, the mass concentration increase of sulfuric acid from 0.2 to 0.8%, aimed to provide the maximum productivity of the process, requires a progressively smaller fraction of NaCl (from 8 to 3%). When the maximum lead leaching zone is imposed (see the red area in Figure 3) on a given response surface, it becomes obvious that their locations are mutually conditioned.

For the case of the preliminary mechanical processing of the tailings, the dependence of the Zn yield on the parameters of the lixiviants was established when the process of the Taylor Series Polynomials type, ° lasted for 15 min, whose formula had the following view (R^2^ = 0.92):

(2) $$\mathrm{Pb}=-61.65+188.19\mathrm{lnNaCl}-283.81{\mathrm{lnH}}_{2}{\mathrm{SO}}_{4}-123.79{\left(\mathrm{lnNaCl}\right)}^{2}-117.57{\left({\mathrm{lnH}}_{2}{\mathrm{SO}}_{4}\right)}^{2}+\ldots \;..+83.30{\mathrm{NaClH}}_{2}{\mathrm{SO}}_{4}+26.70{\left(\mathrm{lnNaCl}\right)}^{3}+28.73{\left({\mathrm{lnH}}_{2}{\mathrm{SO}}_{4}\right)}^{3}+88.23\mathrm{lnNaCl}{\left({\mathrm{lnH}}_{2}{\mathrm{SO}}_{4}\right)}^{2}+\ldots \;+30.49{\left(\mathrm{lnNaCl}\right)}^{2}{\mathrm{lnH}}_{2}{\mathrm{SO}}_{4},$$

**Figure 4:**
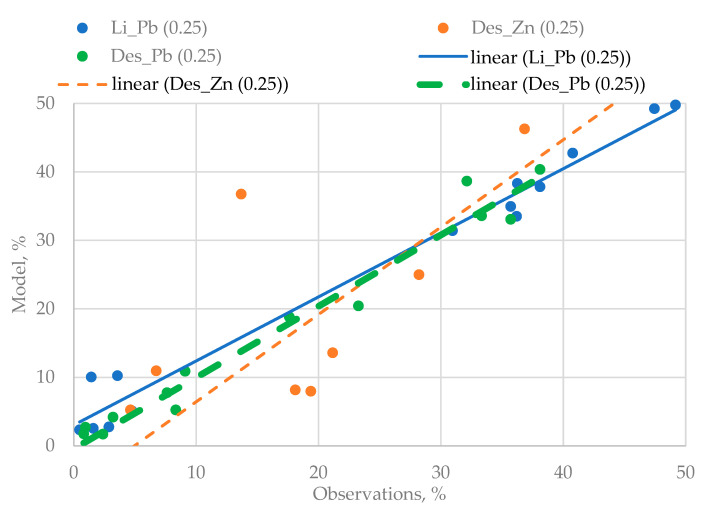
The Q–Q used for three variants of the regression models.

The Q–Q graphs shown in Figure 4 were selected as a “goodness-of-fit” criterion used for checking the quality of three-dimensional models.

The analysis of Figure 4 shows that the quality of the obtained regression equations is very high. The last stage of the experiments is conditioned by the need to identify the mutual influence of the disintegration effect and the agitation leaching duration on the degree of the lead yielded from the technogenic raw materials.

### 3.2. The Mechanical Activation Effect during the Prolonged Leaching of Pb 

**Figure 5:**
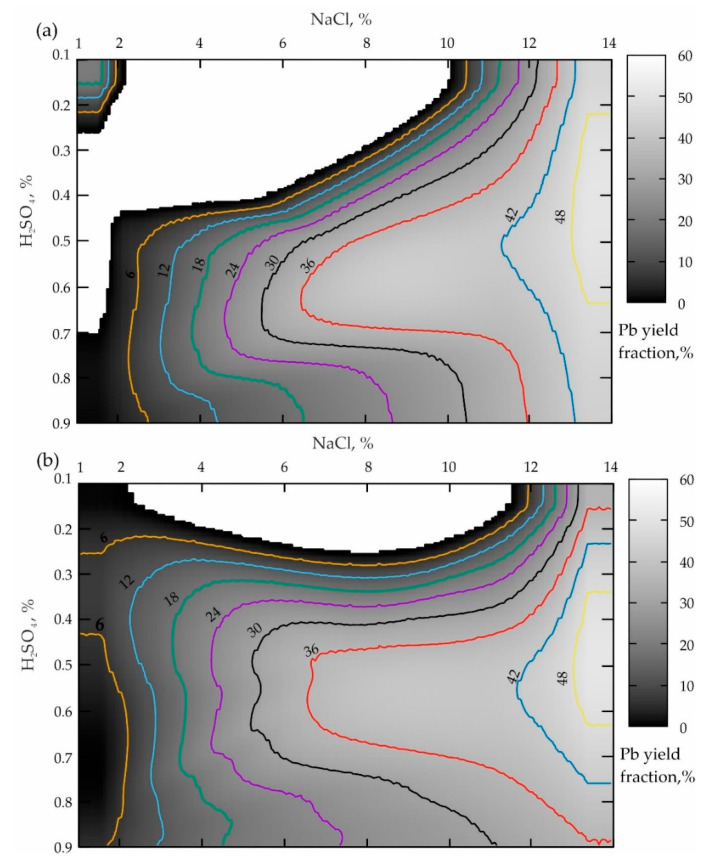
Distribution of the lead yielded from the tailings of the Mizursky factory: (**a**)—agitation leaching of Pb lasting for 60 min; (**b**)—leaching Pb from the preliminary activated tailings at (υ) = 300 rpm lasting for 60 min.

The processing of the corresponding results presented in Table 4 is provided in Figure 5. 

The analysis of the response surface projection provided in Figure 5a allows the conclusion that the NaCl concentration increases from 1 to 14% when the boundary value of H_2_SO_4_ is 0.1%, leading to a monotonous increase in the lead yield in the pulp from 6 to 44% (when NaCl ranges from 10.2 to 14%). The boundary H_2_SO_4_ value of 0.9% increases the lead yield in the pulp from 6 to 43% when NaCl ranges from 2% to 14%. In the case of the optimal sulfuric acid concentration (ranging from 0.5 to 0.72%), starting with NaCl ≥ 6.3%, a zone of the local maximum Pb yield having a minimum value of 36% is also traced, which increases up to 42% when NaCl ≥ 12%. This maximum reaches the highest value of 48% when NaCl ≥ 13.4%.

The analysis of the results presented in Figure 2b establishes the fact that the preliminary activation of the geomaterial (when t = 60 min) results in a similar picture presented in Figure 2a. The main difference is a significant expansion of the local maxima area towards a lower concentration of sulfuric acid (apparently because of an increase in reactivity due to the amorphization of the geomaterials).

The comparison of the two variants of the agitation leaching shown in Figure 2a and Figure 5a, when the process duration is 15 and 60 min, allows the conclusion that the more focused area of the maximum during a shorter leaching time is replaced by a uniformly elongated area directed towards a smaller NaCl concentration. When comparing the two variants of leaching the activated tailings (Figure 2a and Figure 5b) while increasing υ from 300 to 1200 rpm, the sizes of both zones of local maxima increase significantly. And the local minimum area disappears altogether (NaCl ranges from 1 to 12.5%; H_2_SO_4_ varies from 0.5–0.9%; the white area inside the darkest grayscale color indicates the area of zero % of the Pb yield).

## 4. Discussion

The comparison of the results shown in Figure 2b and Figure 3 makes it possible to conclude that the obvious fact is a higher increase in the Zn reactivity as compared to that of Pb after dry mechanical activation of the tailings. The absolute values of the local maximum of the zinc yield are 54%, and in the same conditions, those of Pb are only 36%. 

In this connection, a significant proportion of smithsonite is apparently present in the tailings. The direct leaching of smithsonite with sulfuric acid is known to be represented as follows:

(3) $${\mathrm{ZnCO}}_{3}+H_{2}{\mathrm{SO}}_{4}\to {\mathrm{ZnSO}}_{4}+H_{2}O+{\mathrm{CO}}_{2},$$

In [55], the simple agitation leaching of Zn from the tailings allowed for establishing the fact that this process proceeded quite quickly, while Pb was practically not extracted. When using hydrochloric acid instead of sulfuric acid, Pb was leached more efficiently into the solution, with the yield ratio of Zn and Pb = 90 and 10%, respectively (or 9/1). In our case, the introduction of additional hydrochloric acid into the leaching solution is conditioned by the need to extract both of the metals. Sodium chloride is required for the formation of hydrochloric acid, which in turn can react with Zn and Pb according to the schemes:

(4) $$\mathrm{NaCl}+H_{2}{\mathrm{SO}}_{4}\to {\mathrm{NaHSO}}_{4}+\mathrm{HCl},\;{\mathrm{ZnCO}}_{3}+2\mathrm{HCl}\to {\mathrm{ZnCl}}_{2}+H_{2}O+{\mathrm{CO}}_{2},$$

(5) $$\mathrm{PbO}+2\mathrm{HCl}\to {\mathrm{PbCl}}_{2}+H_{2}O,$$

The area of the maximum Zn zone shown in Figure 3 is 6–7 times higher, and it is located much to the left along the NaCl axis. This can be explained by the fact that at low concentrations of sulfuric acid ranging from 0.1 to 0.35%, all the acid reacts actively according to the reaction (3), which provides a disadvantage for the HCl formation. At an optimal level of H_2_SO_4_ varying from 0.4 to 0.55%, the maximum volumes of sulfuric acid seem to react with NaCl and cause an increase in reaction volumes by the reaction (5). At the same time, to obtain optimal process parameters, the ratio of the Zn and Pb yield must be 2/1, which determines the efficiency improvement of the metal leaching during mechanical activation.

The mutual location of the two zones of the optimal leaching of the metal indicates that zinc reacts at lower concentrations of the reagents that are present in the leaching solution. Lead leaching without mechanical activation is known to be about 23% [101]. Oxide, silicate, or carbonate forms of zinc dissolve more easily in the sulfuric acid solution as compared to lead disulfate [102,103]. The efficiency coefficient of using this acid (according to the (AHP) method in the case of Zn) is the maximum possible (0.709), and in the case of Pb, it is only 0.157. In our case, we managed to achieve 36% or more, while the local maximum area became more focused in the region of optimal concentrations of H_2_SO_4_ and NaCl. The obtained results of the optimum yield values of Zn (54%) and Pb (36–48%) correlated well with the results of the flotation of the lead–zinc ores of the Belousovskaya enrichment factory (Kazakhstan), where the recovery percentage was 58% and 29%, respectively [47]. In the study of Chinese researchers, applying the mechanical activation of sphalerite for 15 min allowed increasing the zinc yield from 5% to 30% [104]. This allows the conclusion that when these two metals are simultaneously present in the tailings, a more soluble and active zinc gains a greater effect during mechanical activation. As a result, proving the effect of increasing the lead yield during preliminary high-energy grinding was impossible in this work.

Without activating the tailings (when H_2_SO_4_ = 1 M, 0.5 M, and 0.25 M), no more than 30% of Zn was extracted in 2 h in another similar study [105]. At the same time, the zinc yields comparable to that presented in our studies (about 50%) could be obtained only in 5 h. Our results show the possibility of obtaining the same metal yield in the case of significantly less leaching time and acid consumption. In the study, during the leaching time of 12 h, the yttrium proportion in the solution was increased to 88% as compared to 48% obtained without activation. In our case, when a leaching duration was 1 h (see Figure 5), the area of the local minimum lead yield significantly decreased (by 50%). Similarly to [57], when the S/L and a sulfuric acid fraction was 200 g/L, a large part of the metal yield during activation in a ball mill was achieved in the first hour. Leaching Cu with sulfuric acid from the ore [59] confirms the process productivity increase accompanied by an increase in the number of rotor rotations. In our case, at high rotor speeds, lead is characterized by the large focus of the second region of the local maximum (48%) towards the optimal values of the sulfuric acid concentration.

Based on the obtained results, the following findings can be formulated:

1. For the first time, the present work has established the fact that during the preliminary dry mechanical activation of the enrichment tailings lasting for 0.25 h, a decrease in the mass NaCl concentration from 14 to 1% when the H_2_SO_4_ concentration decreases from 0.8 to 0.2% occurs, which leads to a zinc yield increase from 12 to 54% by the logarithmic dependence;
2. For the first time, the present study has determined the fact that during the preliminary dry mechanical activation of the enrichment tailings lasting for 0.25 h, a decrease in the mass NaCl concentration from 8 to 5% when the H_2_SO_4_ concentration decreases from 0.38 to 0.51% occurs, increasing the lead yield from 6 to 36% according to the dependence of a complex type;
3. Using a disintegrator to induce an activation effect significantly transforms the response surfaces of the process under study, improving the efficiency of the Pb yield from the enrichment tailings at a lower cost of reagents (expansion of the local maxima areas regardless of the number of rotor rotations);
4. One of the key results of the research is the confirmation of the hypothesis about the presence of a different reactivity when leaching the metals (in the case of Zn, it is several times higher than that in the case of Pb), which is conditioned by the mechanochemical activation of the dry tailings in the disintegrator.

The waste obtained at this stage, in accordance with the author’s approach, must be used in the mine construction as fillers in filling mixtures. Some authors [106] suggest using iron ore tailings (up to 20% by the concrete weight) as an inert filler in a mixture containing cement. If the waste is reactivated in order to improve the properties of the filling mass, as the study [107] mentions, the strength of the samples can be increased two times. Even using the copper tailings as an additional cement material can be environmentally justified [108,109,110]. In our previous studies concerning this type of tailings, the backfilling strength was found to be 8–9 MPa [84]. Other studies [111,112] also indirectly confirm the efficiency of applying high-energy effects to improve the properties of geomaterials. In our case, we propose using an alternative to foaming materials [113,114,115] applied for the formation of filling masses or security structures in the form of high-performance polyethylene filled with an inert filler composition, which should act as enrichment tailings.

## 5. Conclusions

Optimization of the agitation leaching parameters using the mechanical activation of the technogenic geomaterials allows obtaining valuable polymetallic raw materials at lower cost prices (the dump masses are already located on the surface). When introducing “circular economy” mechanisms, the widespread use of disintegrators in order to involve dump masses as an inert filler used for security strips will make waste recycling in mining even more profitable. The main factor that will contribute to this concept implementation is an efficiency increase in the preliminary extraction of the metals due to the fact that if the agitation leaching is short-term, the mechanical activation of the tailings will necessarily enhance the reactivity (and consumption of lixiviants in the leaching solution) of Zn compared with that of Pb. This circumstance should be taken into account when justifying the parameters of the full cycle of the multistage and complex processing of the technogenic raw materials before using it as an inert filler.

The practical significance of applying the disintegrators for implementing the circular waste management concept may consist in the subsequent development of the regulations on applying the mechanical activation effect to technogenic waste for the purpose of increasing the yield of metals and forming security strips.

Further research should be aimed at specifying the types of chemical reactions allowing Pb and Zn leaching in the H_2_SO_4–_NaCl solution for such conditions (based on the mineralogical studies of the samples), as well as searching for rational forms of security strips based on the obtained results. The conclusions are valid only for the geomaterials obtained from the Sadonsky mining district. The main limitations include the ranges of the components present in the leaching solution (based on H_2_SO_4_ = 0.1–0.9%, NaCl = 1–12.5%); the process duration is 0.25–1 h; the S–L ratio is from 1/4 to 1/10.

## Figures and Tables

**Table 1:** The chemical composition of the enrichment tailings.

Component	Content (%)
Pb	0.84 ± 0.06
Zn	0.95 ± 0.06
TiO_2_	0.03 ± 0.001
Al_2_O_3_	0.8 ± 0.04
K_2_O	3.5 ± 0.05
Mn	0.015 ± 0.002
Cu	0.18 ± 0.08
Ag	0.015 ± 0.002
S	1.88 ± 0.15
CaO	1.96 ± 0.15
Fe_2_O_3_	4.4 ± 0.05
SiO_2_	31.4 ± 0.13

**Table 2:** Granulometric composition of tailings.

	Residue on Sieve, mm, %							
Sieve size, mm	1.6	1	0.63	0.40	0.315	0.2	0.16	0.1
Activated tails, %	3.18	4.56	4.12	6.20	9.72	15.46	18.30	24.12
Original tails, %	22.3	11.25	8.62	5.74	4.01	4.26	3.22	2.09

**Table 3:** Type parameters of sets of experiments.

	Type Name of a Group of Experiments				
	Li_Pb(0.25)	Des_Pb(0.25)	Des_Zn(0.25)	Li_Pb(1)	Des_Pb(1)
Metal type	Pb	Pb	Zn	Pb	Pb
t (min)	15	15	15	60	60
Speed (rpm)	-	300	300	-	1200

**Table 4:** Variants of the experiments in each set of groups of the experiments.

N	*h* (H_2_SO_4_)	*h* (NaCl)	S/L	*M_P_*	*m_P_* (H_2_SO_4_)	*m_P_* (NaCl)
	g/L	g/L		g	%	%
1	2	3	9	11	12	13
1	2	20	1/4	250	0.16	1.58
2	6	20	1/4	250	0.47	1.58
3	10	20	1/4	250	0.79	1.58
4	2	160	1/4	250	0.15	11.79
5	6	160	1/4	250	0.44	11.77
6	10	160	1/4	250	0.73	11.75
7	6	90	1/4	250	0.46	6.86
8	6	90	1/7	400	0.50	7.50
9	2	20	1/10	550	0.18	1.80
10	6	20	1/10	550	0.54	1.80
11	10	20	1/10	550	0.90	1.79
12	2	160	1/10	550	0.17	13.40
13	6	160	1/10	550	0.50	13.38
14	10	160	1/10	550	0.83	13.36

**Table 5:** Results of the five sets of experiments on the efficiency of leaching the metals from technogenic raw materials.

N	Experiment Group Names				
	Li_Pb(0.25)	Des_Pb(0.25)	Des_Zn(0.25)	Li_Pb(1)	Des_Pb(1)
1	2	3	4	5	6
1	1.43	0.81	19.37	17.14	1.43
2	1.59	8.34	88.72	3.12	5.39
3	0.48	0.95	56	1.43	0.86
4	36.19	17.62	6.74	24.76	3.33
5	40.77	9.1	21.16	41.78	38.71
6	38.1	2.38	28.21	37.14	37.14
7	47.45	32.12	53.34	24.38	34.99
8	49.17	33.33	36.84	40.83	45
9	3.57	3.21	50.53	3.57	2.98
10	2.87	7.62	88.04	2.08	5.58
11	4.76	2.62	61.05	1.79	6.67
12	30.95	35.71	4.63	46.43	36.9
13	36.25	23.26	18.1	50.3	51.7
14	35.71	38.1	13.68	44.05	38.1

## Data Availability

The data presented in this study are available from the corresponding authors upon reasonable request.

## References

[1] Chen X., Zhao B., Shuai C., Qu S., Xu M. (2022). Global spread of water scarcity risk through trade. Resour. Conserv. Recycl..

[2] Ghorbani Y.G.T., Nwaila S.E., Zhang J.E., Bourdeau M., Cánovas J., Arzua N., Nadat N. (2023). Moving towards deep under-ground mineral resources: Drivers, challenges and potential solutions. Resour. Policy.

[3] Iddagoda A., Manta O., Dissanayake H., Abeysinghe R., Perera D. (2023). Combatting Environmental Crisis: Green Orientation in the Sri Lanka Navy. J. Risk Financ. Manag..

[4] Qu Q., Guo H., Yuan L., Shen B., Yu G., Qin J. (2022). Rock Mass and Pore Fluid Response in Deep Mining: A Field Monitoring Study at Inclined Longwalls. Minerals.

[5] Dzhioeva A.K. (2022). Prospects for mining ecologization to reduce harmful emissions into the atmosphere. Ugol.

[6] Feng Y., Hu J., Afshan S., Irfan M., Hu M., Abbas S. (2023). Bridging resource disparities for sustainable development: A comparative analysis of resource-rich and resource-scarce countries. Resour. Policy.

[7] Yu L., Yu P.S., Duan Y., Qiao H. (2022). A resource scheduling method for reliable and trusted distributed composite services in cloud environment based on deep reinforcement learning. Front. Genet..

[8] Agboola O., Babatunde D.E., Isaac Fayomi O.S., Yahaya A., Mamudu O.A. (2020). A review on the impact of mining operation: Monitoring, assessment and management. Results Eng..

[9] Khayrutdinov M.M., Kaung P.A., Chzho Z.Y., Tyulyaeva Y.S. (2022). Ensuring Environmental Safety in the Implementation of the Resource-renewable Technologies. Bezop. Tr. V Promyshlennosti.

[10] Cacciuttolo C., Atencio E. (2022). Past, Present, and Future of Copper Mine Tailings Governance in Chile (1905–2022): A Review in One of the Leading Mining Countries in the World. Int. J. Environ. Res. Public Health.

[11] Brigida V.S., Golik V.I., Dmitrak Y.V., Gabaraev O.Z. (2019). The impact of situational geomechanical conditions influence to improving of the drainage rock-mass caved. Proc. Tula States Univ. -Sci. Earth.

[12] Mensah M.K., Drebenstedt C., Hoth N., Ola I.F., Okoroafor P.U., Wiafe E.D. (2023). Artisanal gold mine spoil types within a com-mon geological area and their variations in contaminant loads and human health risks. Environ. Monit. Assess..

[13] Chen T., Wen X.-C., Zhang L.-J., Tu S.-C., Tu S.-C., Zhang J.-H., Sun R.-N., Yan B. (2022). The geochemical and mineralogical controls on the release characteristics of potentially toxic elements from lead/zinc (Pb/Zn) mine tailings. Environ. Pollut..

[14] Qi C., Wu M., Liu H., Liu X., Lin Z. (2023). Machine learning exploration of the mobility and environmental assessment of toxic elements in mining-associated solid wastes. J. Clean. Prod..

[15] Zachinskis A., Grechenkov J., Butanovs EPlatonenko A., Piskunov S., Popov A.I., Purans J., Bocharov D. (2023). Ir impurities in α- and β-Ga2O3 and their detrimental effect on p-type conductivity. Sci. Rep..

[16] Zhong X., Chen Z., Ding K., He Z., Qiu R. (2023). Heavy metal contamination affects the core microbiome and assembly process-es in metal mine soils across. Eastern China. J. Hazard. Mater..

[17] Guo Q., Peng H., Hong B., Yao H., Zhu Y., Ding H., An N., Hong Y. (2021). Variations of methane stable isotopic values from an Alpine peatland on the eastern Qinghai-Tibetan Plateau. Acta Geochim..

[18] Yao H., Peng H., Hong B., Guo Q., Ding H., Hong Y., Zhu Y., Cai C., Chi J. (2022). Environmental Controls on Multi-Scale Dy-namics of Net Carbon Dioxide Exchange from an Alpine Peatland on the Eastern Qinghai-Tibet Plateau. Front. Plant Sci..

[19] Gabasiane T.S., Danha G., Mamvura T.A., Mashifana T., Dzinomwa G. (2021). Environmental and Socioeconomic Impact of Copper Slag—A Review. Crystals.

[20] Adrianto L.R., Pfister S. (2022). Prospective environmental assessment of reprocessing and valorization alter-natives for sulfidic cop-per tailings. Resour. Conserv. Recycl..

[21] Li Z., Zhang Y., Luo T., Xia P., Mu H., Sun P., Wang X., Wang J. (2023). In-Situ Leaching Mining Technique for Deep Bauxite Extrac-tion and the Countermeasures for Water Pollution Prevention: An Example in the Ordos Basin, China. Water.

[22] Rybak J., Adigamov A., Kongar-Syuryun C., Khayrutdinov M., Tyulyaeva Y. (2021). Renewable-resource technologies in mining and metallurgical enterprises providing environmental safety. Minerals.

[23] Devaraj V., Mangottiri V., Balu S. (2023). Sustainable utilization of industrial wastes in controlled low-strength materials: A review. Environ. Sci. Pollut. Res..

[24] Yu H. (2023). Mining waste: Curb risks to people and the environment. Nature.

[25] Gökelma M., Vallejo-Olivares A., Tranell G. (2021). Characteristic properties and recyclability of the aluminum fraction of MSWI bottom ash. Waste Manag..

[26] Shaforostova E.N., Kosareva-Volod’ko O.V., Belyankina O.V., Solovykh D.Y., Sazankova E.S., Sizova E.I., Adigamov D.A. (2023). A Tailing Dump as Industrial Deposit; Study of the Mineralogical Composition of Tailing Dump of the Southern Urals and the Possibility of Tailings Re-Development. Resources.

[27] Zolfaghari S., Mostofinejad D., Fantuzzi N., Luciano R., Fabbrocino F. (2023). Experimental evaluation of FRP-concrete bond using externally-bonded reinforcement on grooves (EBROG) method. Compos. Struct..

[28] Malyukova L.S., Martyushev N.V., Tynchenko V.V., Kondratiev V.V., Bukhtoyarov V.V., Konyukhov V.Y., Bashmur K.A., Panfilova T.A., Brigida V. (2023). Circular Mining Wastes Management for Sustainable Production of *Camellia sinensis* (L.) O. Kuntze. Sustainability.

[29] Aleid G.M., Alshammari A.S., Ahmad A.R.D., Hussain F., Oh S.-E., Ahmad A., Ibrahim M.N.M., Umar K. (2023). Advancement in Microbial Fuel Cells Technology by Using Waste Extract as an Organic Substrate to Produce Energy with Metal Removal. Processes.

[30] Moyo L.B., Simate G.S., Mamvura T.A. (2022). Magnesium recovery from ferrochrome slag: Kinetics and possible use in a circular economy. Heliyon.

[31] Gómez-Sanabria A., Kiesewetter G., Klimont Z., Schoepp W., Haberl H. (2022). Potential for future reductions of global GHG and air pollutants from circular waste management systems. Nat. Commun..

[32] Al-Shawabkeh A.F., Thalji M.O., Al-Rousan T.M. (2022). Using recycled plastic waste to improve the performance of hot-mix asphalt. Proc. Inst. Civ. Eng. Waste Manag. Res..

[33] Singh R., Khan S., Dsilva J. (2022). A framework for assessment of critical factor for circular economy practice implementation. J. Mod-el. Manag..

[34] Zhanbayev R.A., Yerkin A.Y., Shutaleva A.V., Irfan M., Gabelashvili K., Temirbaeva G.R., Chazova I.Y., Abdykadyrkyzy R. (2023). State asset management paradigm in the quasi-public sector and environmental sustainability: Insights from the Republic of Kazakhstan. Front. Environ. Sci..

[35] Geissdoerfer M., Savaget P., Bocken N.M.P., Hultink E.J. (2017). The Circular Economy—A new sustainability paradigm?. J. Clean. Prod..

[36] Klyuev R.V., Yegorova E.V., Bosikov I.I., Tsidaev B.S. (2018). Evaluation of use of effective technologies for increasing sustainable development of natural and technical system of oil and gas complex. Sustain. Dev. Mt. Territ..

[37] Fantuzzi N. (2023). Novel approaches for the multiscale analysis of composite materials and structures. Compos. Part C Open Acc..

[38] Zhanbayev R.A., Irfan M., Shutaleva A.V., Maksimov D.G., Abdykadyrkyzy R., Filiz Ş. (2023). Demoethical Model of Sustainable Development of Society: A Roadmap towards Digital Transformation. Sustainability.

[39] Saldana M., Galvez E., Robles P., Castillo J., Toro N. (2022). Copper Mineral Leaching Mathematical Models—A Review. Materials.

[40] Palaniandy S. (2015). Impact of mechanochemical effect on chalcopyrite leaching. Int. J. Miner. Process..

[41] Zhai J.H., Wang H.B., Chen P., Hu Y., Sun W. (2020). Recycling of iron and titanium resources from early tailings: From fundamental work to industrial application. Chemosphere.

[42] Golik V.I., Razorenov Y.U.I., Brigida V.S., Burdzieva O.G. (2020). Mechanochemical technology of metal mining from enriching tails. Bull. Tomsk. Polytech. Univ. Geo Assets Eng..

[43] Gümüşsoy A., Başyiğit M., Uzun Kart E. (2023). Economic potential and environmental impact of metal recovery from copper slag flotation tailings. Resour. Policy.

[44] Whitworth A., Zhou F.J., Vaughan J., Southam G., Van der Ent A., Nkrumah P.N., Ma X., Parbhakar-Fox A. (2022). Review on metal extraction technologies suitable for critical metal recovery from mining and processing wastes. Miner. Eng..

[45] Golik V.I., Khasheva Z.M., Petrovich S.L. (2015). Economical efficiency of utilization of allied mining enterprises waste. Soc. Sci. (Pak.).

[46] Zhou F., Xiao Y., Guo M., Tang Y., Zhang W., Qiu R. (2021). Selective Leaching of Rare Earth Elements from Ion-Adsorption Rare Earth Tailings: A Synergy between CeO_2_ Reduction and Fe/Mn Stabilization. Environ. Sci. Technol..

[47] Seksenova N., Bykov R., Mamyachenkov S., Daumova G., Kozhakanova M. (2021). Optimization of Conditions for Processing of Lead–Zinc Ores Enrichment Tailings of East Kazakhstan. Metals.

[48] Derkaoui I., Achehboune M., Eglitis R.I., Popov A.I., Rezzouk A. (2023). Overview of the Structural, Electronic and Optical Properties of the Cubic and Tetragonal Phases of PbTiO3 by Applying Hubbard Potential Correction. Materials.

[49] Komashchenko V.I., Vorobyov E.D., Razorenov Y.I. (2017). Extraction of metals when recycling enrichment of ores. Bull. Tomsk. Polytech. Univ. Geo Assets Eng..

[50] Gerasimenko T.E., Rubayeva I.O., Maksimov R.N., Vasiliev V.V. (2023). Peculiarities of poly disperse particle interaction in gold micro dispersions flotation processes. Sustain. Dev. Mt. Territ..

[51] Lakshmanan V.I., Sridhar R., Chen J., Halim M.A. (2017). A Mixed-Chloride Atmospheric Leaching Process for the Recovery of Base Metals from Sulphide Materials. Trans. Indian Inst. Met..

[52] Kyaw Z.Y., Htet Z.O., Shekhirev D.V., Goryachev B.E. (2023). The effect of ferrous sulfate, sodium sulfide and their mixtures on the flotation of sphalerite in the alkaline medium. Sustain. Dev. Mt. Territ..

[53] Farhan A., Zahid M., Tahir N. (2023). Investigation of boron-doped graphene oxide anchored with copper sulphide flowers as visible light active photocatalyst for methylene blue degradation. Sci. Rep..

[54] Basturkcua H., Acarkan N., Gock E. (2017). The role of mechanical activation on atmospheric leaching of a lateritic nickel ore. Int. J. Miner. Process..

[55] Hussaini S., Kursunoglu S., Top S., Ichlas Z.T., Kaya M. (2021). Testing of 17-different leaching agents for the recovery of zinc from a carbonate-type Pb-Zn ore flotation tailing. Miner. Eng..

[56] Li X., Li W., Gao Y., Tian G. (2022). Effect of Mechanical Activation on the Leaching Process of Rare Earth Metal Yttrium in Deep Eutectic Solvents. Appl. Sci..

[57] Palaniandy S., Azizli K.A.M., Hussin H., Hashim S.F.S. (2007). Study on mechanochemical effect of silica for short grinding period. Int. J. Miner. Process..

[58] Zheng X.H., Lu W.G., Cao H.B., Cai N., Li Q., Kang F., Sun Z. (2021). Leaching of valuable metals from nickel sulfide ores by mechanical activation. Chin. J. Process Eng..

[59] Minagawa M., Hisatomi S., Kato T., Granata G., Tokoro C. (2018). Enhancement of copper dissolution by mechanochemical activation of copper ores: Correlation between leaching experiments and DEM simulations. Adv. Powder Technol..

[60] Mulenshi J., Chelgani S.C., Rosenkranz J. (2021). Mechanochemical Treatment of Historical Tungsten Tailings: Leaching While Grinding for Tungsten Extraction Using NaOH. Sustainability.

[61] Nkuna R., Ijoma G.N., Matambo T.S., Chimwani N. (2022). Accessing Metals from Low-Grade Ores and the Environmental Impact Considerations: A Review of the Perspectives of Conventional versus Bioleaching Strategies. Minerals.

[62] Ming Z., Ya-Na L., Shu-Fa Z., Juan M., Tie-You D. (2012). Removal of Cu, Zn and Pb from mine tailings by bioleaching: Effects of initial pH. Int. J. Environ. Stud..

[63] Chen T., Lei C., Yan B., Xiao X. (2014). Metal recovery from the copper sulfide tailing with leaching and fractional precipitation technology. Hydrometallurgy.

[64] Ardashkin I.B., Yakovlev A.N., Martyushev N.V. (2014). Evaluation of the resource efficiency of foundry technologies: Methodological aspect. Adv. Mater. Res..

[65] Abkhoshk E., Jorjani E., Al-Harahsheh M.S., Rashchi F., Naazeri M. (2014). Review of the hydrometallurgical processing of non-sulfide zinc ores. Hydrometallurgy.

[66] Yang X., Honaker R.Q. (2020). Leaching Kinetics of Rare Earth Elements from Fire Clay Seam Coal. Minerals.

[67] Crane R.A., Sapsford D.J. (2018). Towards Greener Lixiviants in Value Recovery from Mine Wastes: Efficacy of Organic Acids for the Dissolution of Copper and Arsenic from Legacy Mine Tailings. Minerals.

[68] Abdel-Aal E. (2000). Kinetics of sulfuric acid leaching of low-grade zinc silicate ore. Hydrometallurgy.

[69] Li Q., Zhang B., Min X., Shen W. (2013). Acid leaching kinetics of zinc plant purification residue. Trans. Nonferrous Met. Soc. China.

[70] Chen A., Zhao Z., Jia X., Long S., Huo G., Chen X. (2009). Alkaline leaching Zn and its concomitant metals from refractory hemimorphite zinc oxide ore. Hydrometallurgy.

[71] Chipakwe V., Karlkvist T., Rosenkranz J., Chelgani S.C. (2022). Beneficial effects of a polysaccharide-based grinding aid on magnetite flotation: A green approach. Sci. Rep..

[72] Ou Z., Li J., Wang Z. (2015). Application of mechanochemistry to metal recovery from second-hand resources: A technical overview. Environ. Sci. Process. Impacts.

[73] Beyer M.K., Clausen-Schaumann H. (2005). Mechanochemistry: The mechanical activation of covalent bonds. Chem. Rev..

[74] Gaesenngwe G., Mamvura T., Danha G., Sibanda V. (2021). A comparative study on the comminution behavior of diorite rocks. Heliyon.

[75] Juhász A.Z. (1998). Aspects of mechanochemical activation in terms of comminution theory. Colloids Surf. A Physicochem. Eng. Asp..

[76] Li Y., Wang B., Xiao Q., Lartey C., Zhang Q. (2017). The mechanisms of improved chalcopyrite leaching due to mechanical activation. Hydrometallurgy.

[77] Wang Z., Chu H., Wang J., Feng E., Zhang Y., Lyu X. (2022). Mechanical activation of siliceous tailings and its application as cement admixtures. Miner. Eng..

[78] Kongar-Syuryun C., Aleksakhin A., Khayrutdinov A., Tyulyaeva Y. (2021). Research of rheological characteristics of the mixture as a way to create a new backfill material with specified characteristics. Mater. Today Proc..

[79] Wang L., Wei Y., Lv G., Liao L., Zhang D. (2019). Experimental Studies on Chemical Activation of Cementitious Materials from Smelting Slag of Copper and Nickel Mine. Materials.

[80] Zhang Q., Yao B., Fan X., Li X., Wang L. (2023). A failure criterion for shale considering the anisotropy and hydration based on the shear slide failure model. Int. J. Min. Sci. Technol..

[81] de Moraes T.M.R.P., Neto O.D.M.M., Lucena A.E.D.F.L., Lucena L.D.F.L., Nascimento M.S. (2023). Viability of Asphalt Mixtures with Iron Ore Tailings as a Partial Substitute for Fine Aggregate. Transp. Res. Rec..

[82] Zhang J., Liu J., Li C., Jin Y., Nie Y., Li J. (2009). Comparison of the fixation effects of heavy metals by cement rotary kiln co-processing and cement based solidification/stabilization. J. Hazard. Mater..

[83] Marsh A.T.M., Yue Z., Dhandapani Y., Adu-Amankwah S., Bernal S.A. (2022). Influence of limestone addition on sodium sulphate activated blast furnace slag cements. Constr. Build Mater..

[84] Golik V.I., Dmitrak Y.V., Brigida V.S. (2020). Impact of duration of mechanochemical activation on enhancement of zinc leaching from polymetallic ore tailings. Nauk. Visnyk Natsionalnoho Hirnychoho Universytetu.

[85] Klyuev R., Fomenko O., Gavrina O., Turluev R., Marzoev S. (2021). Energy indicators of drilling machines and excavators in mountain territories. Adv. Intell. Syst. Comput..

[86] Horry M.J., Chakraborty S., Pradhan B., Shulka N., Almazroui M. (2023). Two-Speed Deep-Learning Ensemble for Classification of Incremental Land-Cover Satellite Image Patches. Earth Syst. Environ..

[87] Pradhan B., Jena R., Talukdar D., Mohanty M., Sahu B.K., Raul A.K., Abdul Maulud K.N. (2022). A New Method to Evaluate Gold Mineralization-Potential Mapping Using Deep Learning and an Explainable Artificial Intelligence (XAI) Model. Remote Sens..

[88] Trzepieciński T., Najm S.M. (2022). Application of Artificial Neural Networks to the Analysis of Friction Behavior in a Draw bead Profile in Sheet Metal Forming. Materials.

[89] Ullah H., Fiza M., Zahoor Raja M.A., Shoaib M., Al-Mekhlafi S.M. (2022). Intelligent Computing of Levenberg-Marquard Technique Backpropagation Neural Networks for Numerical Treatment of Squeezing Nano fluid Flow between Two Circular Plates. Math. Probl. Eng..

[90] Chehreh Chelgani S., Nasiri H., Tohry A., Heidari H.R. (2023). Modeling industrial hydrocyclone operational variables by SHAP-CatBoost—A “conscious lab” approach. Powder Technol..

[91] Al-Shawabkeh A.F., Elimat Z.M., Abushgair K.N. (2023). Effect of non-annealed and annealed ZnO on the optical properties of PVC/ZnO nanocomposite films. J. Thermoplast. Compos. Mater..

[92] Monari G., Galeotti M., Matteini M., Salvadori B., Stifanese R., Traverso P., Vettori S., Letardi P. (2023). Protective treatments for copper alloy artworks: Preliminary studies of sodium oxalate and limewater effectiveness against bronze disease. Environ. Sci. Pollut. Res..

[93] Romanenkov Y., Pronchakov Y., Zieiniiev T. (2020). Robust estimation of the area of adequacy of forecasting one parameter model of exponential smoothing. East. -Eur. J. Enterp..

[94] Liu X., Aldrich C. (2022). Assessing the Influence of Operational Variables on Process Performance in Metallurgical Plants by Use of Shapley Value Regression. Metals.

[95] Vorobieva I.A., Gvishiani A.D., Dzeboev B.A., Dzeranov B.V., Barykina Y.V., Antipova A.O. (2022). Nearest Neighbor Method for Discriminating Aftershocks and Duplicates When Merging Earthquake Catalogs. Front. Earth Sci..

[96] Wang S., Liu K., Wang S. (2022). Three-dimensional stochastic distribution characteristics of void fraction in longwall mining-disturbed overburden. Bull. Eng. Geol. Environ..

[97] Sheresheva M.Y., Kolesnik N.A. (2011). Stochastic perspective of industrial distribution network processes. Industrial Marketing Manag..

[98] Pashkov E.N., Martyushev N.V., Ponomarev A.V. (2014). An investigation into autobalancing devices with multireservoir system. IOP Conf. Ser. Mater. Sci. Eng..

[99] Brigida V.S., Golik V.I., Klyuev R.V., Sabirova L.B., Mambetalieva A.R., Karlina Y.I. (2023). Efficiency Gains When Using Activated Mill Tailings in Underground Mining. Metallurgist.

[100] Brigida V.S., Zinchenko N.N. (2014). Methane Release in Drainage Holes Ahead of Coal Face. J. Min. Sci..

[101] Hu H., Zhang Q., Yuan W., Li Z., Zhao Y., Gu W. (2019). Efficient Pb removal through the formations of (basic) carbonate precipitates from different sources during wet stirred ball milling with CaCO_3_. Sci. Total Environ..

[102] Kursunoglu S., Kursunoglu N., Hussaini S., Kaya M. (2021). Selection of an appropriate acid type for the recovery of zinc from a flotation tailing by the analytic hierarchy process. J. Clean. Prod..

[103] Kondratiev V.V., Karlina A.I., Guseva E.A., Konstantinova M.V., Gorovoy V.O. (2018). Structure of Enriched Ultradisperse Wastes of Silicon Production and Concretes Modified by them. IOP Conf. Ser. Mater. Sci. Eng..

[104] Zhang C., Zhao Y. (2009). Mechanochemical leaching of sphalerite in an alkaline solution containing lead carbonate. Hydrometallurgy.

[105] Álvarez M.L., Méndez A., Rodríguez-Pacheco R., Paz-Ferreiro J., Gascó G. (2021). Recovery of Zinc and Copper from Mine Tailings by Acid Leaching Solutions Combined with Carbon-Based Materials. Appl. Sci..

[106] Fontes W.C., Mendes J.C., Da Silva S.N., Peixoto R.A.F. (2016). Mortars for laying and coating produced with iron ore tailings from tailing dams. Constr. Build. Mater..

[107] Tole I., Habermehl-Cwirzen K., Rajczakowska M., Cwirzen A. (2018). Activation of a Raw Clay by Mechanochemical Process—Effects of Various Parameters on the Process Efficiency and Cementitious Properties. Materials.

[108] Li S., Yu Z., Yu H., Wang X. (2022). The Recent Progress China Has Made in High-Concentration Backfill. Sustainability.

[109] Kondrakhin V.P., Martyushev N.V., Klyuev R.V., Sorokova S.N., Efremenkov E.A., Valuev D.V., Mengxu Q. (2023). Mathematical Modeling and Multi-Criteria Optimization of Design Parameters for the Gyratory Crusher. Mathematics.

[110] Araujo F.S.M., Taborda-Llano I., Nunes E.B., Santos R.M. (2022). Recycling and Reuse of Mine Tailings: A Review of Advancements and Their Implications. Geosciences.

[111] Nemarov A.A., Lebedev N.V. (2016). Theoretical and experimental research of parameters of pneumatic aerators and elementary cycle flotation. Int. J. Appl. Eng. Res..

[112] Marinin M.A., Karasev M.A., Pospekhov G.B., Pomortseva A.A., Sushkova V.I. (2023). Engineering and geological parameters for heap leaching of gold from low-grade sandy clay ores: A feasibility study. Mining Inf. Anal. Bull..

[113] Na H., Lv G., Wang L., Liao L., Zhang D., Guo L., Li W. (2021). A new expansion material used for roof-contacted filling based on smelting. Sci. Rep..

[114] Gutarevich V.O., Martyushev N.V., Klyuev R.V., Kukartsev V.A., Kukartsev V.V., Iushkova L.V., Korpacheva L.N. (2023). Reducing Oscillations in Suspension of Mine Monorail Track. Appl. Sci..

[115] Kondratiev V.V., Karlina A.I., Guseva E.A., Konstantinova M.V., Kleshnin A.A. (2018). Processing and Application of Ultra disperse Wastes of Silicon Production in Construction. IOP Conf. Ser. Mater. Sci. Eng..

